# Inhibition of E2F1/CDK1 Pathway Attenuates Neuronal Apoptosis *In Vitro* and Confers Neuroprotection after Spinal Cord Injury *In Vivo*

**DOI:** 10.1371/journal.pone.0042129

**Published:** 2012-07-25

**Authors:** Junfang Wu, Giorgi Kharebava, Chunshu Piao, Bogdan A. Stoica, Michael Dinizo, Boris Sabirzhanov, Marie Hanscom, Kelsey Guanciale, Alan I. Faden

**Affiliations:** Department of Anesthesiology & Center for Shock, Trauma and Anesthesiology Research (STAR), University of Maryland School of Medicine, Baltimore, Maryland, United States of America; Hertie Institute for Clinical Brain Research, University of Tuebingen, Germany

## Abstract

Apoptosis of post-mitotic neurons plays a significant role in secondary tissue damage following traumatic spinal cord injury (SCI). Activation of E2F1-dependent transcription promotes expression of pro-apoptotic factors, including CDK1; this signal transduction pathway is believed to represent an important mechanism for the physiological or pathological neuronal cell death. However, a specific role for this pathway in neuronal apoptosis induced by SCI has not yet been reported. Here we demonstrate up-regulation of the E2F1/CDK1 pathway that is associated with neuronal apoptosis following impact SCI in rats. Expression of E2F1 and CDK1 were robustly up-regulated as early as 15 min after injury and sustained until 3 days post-injury. CDK1 activity and E2F1 downstream targets bim and c-Myb were significantly increased after SCI. Activation of E2F1/CDK1 signaling also was associated with death of neurons *in vitro*; this was attenuated by shRNA knockdown or pharmacological inhibition of the E2F1/CDK1 pathway. CR8, a novel and potent CDK1 inhibitor, blocked apoptosis of primary cortical neurons at low-micromolar concentrations. Moreover, SCI-induced up-regulation of E2F1/CDK1 and associated neuronal apoptosis was significantly attenuated by systemic injection of CR8 (1 mg/kg, i.p.) at 5 min after injury. CR8 significantly decreased posttraumatic elevation of biochemical markers of apoptosis, such as products of caspase-3 and α–fodrin cleavage, as well as neuronal cell death, as indicated by TUNEL staining. Importantly, CR8 treatment also increased the number of surviving neurons at 5 weeks after injury. Together, these findings indicate that activation of the E2F1/CDK1 pathway contributes to the pathophysiology of SCI and that selective inhibition of this signaling cascade may represent an attractive therapeutic strategy.

## Introduction

Spinal cord injury (SCI) initiates a process of delayed neuronal cell death that contributes to posttraumatic tissue damage and related functional deficits. Many of these neurons undergo apoptosis in response to cell cycle activation [1]–[3]. These changes may reflect decreased survival signals or activation of pro-apoptotic transcription factors and protein kinases [4]–[7] that are believed to trigger apoptotic pathways [8]–[10]. Although intrinsic or extrinsic signals mediating neuronal apoptosis has been studied extensively [11], the detailed mechanisms leading to apoptotic death of neurons following SCI remains to be clarified.

Increased expression of cell cycle related proteins in post-mitotic neurons occurs in many acute and chronic neurodegenerative disorders [12]–[14], including neurotrauma [1], [15]–[16], [11]. Cell cycle activation in neurons was initially described using sympathetic neurons subjected to nerve growth factor (NGF) deprivation [17]. Subsequent studies demonstrated that cyclin D1 elevation in these cells leads to activation of cyclin dependent kinase 4/6 (CDK4/6) followed by increased transcription of E2F pro-apoptotic targets [18]. The E2F1 transcription factor has been widely studied for its role in cell proliferation and apoptosis [19]–[21]. These two seemingly opposite properties are believed to reflect cellular context [21]. For example, in post-mitotic cerebellar granule neurons activation of E2F1 mediates apoptosis through CDK1 expression [22]–[23]. Correspondingly, selective CDK inhibitors like roscovitine, which targets CDK1, but not CDK4 and 6, reduce neuronal apoptosis [24]–[25] and improve behavioral outcomes in traumatic brain injury and focal cerebral ischemia models [26]–[28]. Although previous research has implicated the E2F1/CDK1 pathway in neuronal apoptosis [22], the role of this signaling in neuronal death following SCI has not been addressed.

We provide first evidence for the up-regulation of the E2F1 transcription factor and its target genes, CDK1 and cyclin A after SCI. We show that CDK1 up-regulation and elevation of its co-activator cyclin B1 is accompanied by neuronal apoptosis in injured spinal cord. We also demonstrate that molecular or pharmacological targeting of E2F1/CDK1 signaling *in vitro* is neuroprotective, and that inhibition of this pathway *in vivo* by CR8, a potent and selective purine analogue CDK inhibitor, reduces neuronal cell death.

## Materials and Methods

### Antibodies and reagents

The plasmids encoding E2F1 and CDK1 were obtained from Addgene (#24225 and #1888, respectively). PCDNA3.1 and EF1βLacZ have been described previously [29]. 27 mer siRNA duplexes for human E2F1 (ID 1869), trilencer-27 universal scrambled negative control siRNA duplex (SR30004), constructs expressing 29 mer shRNAs against Rat Cdc2 and E2F1 in pGFP-V-RS vectors were obtained from Origene Technologies Inc. Rat Neuron Nucleofector® Kit (VPG-1003) was purchased from Lonza. ApopTag® Fluorescein/Red detection kit was purchased from Millipore. The following antibodies and reagents were obtained from commercial sources: polyclonal rabbit anti-CDK1, cyclin B1, cyclin A, Bim, and c-Myb (Santa Cruz Biotechnology), mouse anti-CDK1 (Santa Cruz Biotechnology), mouse anti-E2F1 (BD pharmingenTM), rabbit anti-phospho(Ser)-CDK substrate, rabbit anti-cleaved caspase 3 (Cell Signaling Technology), anti-pS54-n-myc (Bethyl Laboratories Inc), rabbit anti-β-galactosidase (MP Biomedicals), monoclonal mouse anti-GAPDH and NeuN (Chemicon), mouse anti-fodrin (Affinity Research Products), mouse anti-APC (CC1, Abcam), mouse anti-CD11b (OX42, Serotec), Hoechst 33258 and other reagents and supplies (Sigma). Roscovitine and CR8 were obtained from Tocris Bioscience.

### Spinal cord injury and drug administration

Adult male Sprague-Dawley rats weighing 275–325 g were subjected to an incomplete contusive SCI [30]. In brief, rats were anesthetized with sodium pentobarbital (65 mg/kg i.p.); laminectomy was performed at vertebral level T8 and the spinal cord was subjected to impact trauma by dropping a 10-g weight from a height of 2.5 cm through a fiberglass guide tube as previously detailed [30]. After SCI, rats were maintained on highly absorbent bedding with their urine manually expressed twice daily until a reflex bladder was established (10–14 d after SCI). Food and water was provided ad libitum. Animals in sham group received a laminectomy without weight drop. All procedures were reviewed and approved by the University of Maryland School of Medicine Animal Care and Use Committee.

After SCI, rats were assigned to a treatment group according to a randomized block experimental design. The number of rats (each n = 4/group) at various time points in each study is also indicated in the figures legends. CR8 was dissolved in sterile saline and administered intraperitoneally, once daily beginning 3 h post-injury and continuing for 7 days. Groups of rats (each n = 6–8/group) received 1 mg/kg CR8 or the equivalent volume of saline. This dose of CR8 was based on the results obtained from pilot studies *in vitro* and *in vivo*. More specifically, anti-apoptotic concentrations of CR8 in cultured cortical neurons were similar to that of flavopiridol, a potent pan-CDK inhibitor. Separate groups of rats (each n = 4/group) received intra-peritoneal injection of CR8 (1 mg/kg) or equal volume of saline, 5 min following injury. Rats were sacrificed for western blot analysis or histology study at 5 or 24 h post-injury.

### Western blotting

Rat spinal cord tissue (5 mm) centered on the injury site was obtained at 15 min, 2 h, 5 h or 1 d, 3 d, or 7 d post injury, with n = 4 rats per time point with an additional 4 laminectomy-only controls. The samples were lysed in radioimmunoprecipitation assay (RIPA) buffer (Sigma) supplemented with 100 mM phenylmethylsulfonyl fluoride, 1x protease inhibitor cocktail, phosphatase inhibitor cocktail II and III (Sigma), then homogenized and sonicated [31]. After centrifugation at 20,600×g for 20 min, protein concentrations in supernatant were determined by the Pierce BCA method (Thermo Scientific). Normalized protein samples were denatured in LDS loading buffer. Each sample was from a different subject and run in an individual lane on 4 to 12% NuPAGE Novex Bis-Tris gradient gels (Invitrogen), and then transferred to nitrocellulose membranes (Invitrogen). After blocking in 5% nonfat milk for 1 h at room temperature, membranes were probed with indicated primary antibodies overnight at 4°C followed by horseradish peroxidase-conjugated secondary antibodies (GE Healthcare) for 1 h at room temperature. The immunocomplexes were then visualized using SuperSignal West Dura Extended Duration Substrate (Thermo Scientific), and quantified by band densitometry of scanned films using the Gel-Pro Analyzer program (Media Cybernetics, Inc.) in the linear detection range. Each sample was repeatedly run three times using the same blot and a pooled average was taken. The error bars in the western blot quantification reflect variance across subjects and repeated runs of the same blots.

### Tissue processing and histopathology

At specific times after injury, rats were anesthetized and transcardially perfused with saline followed by 4% paraformaldehyde (PFA) in PBS. The dissected spinal cords were post-fixed for 2 hours and cryoprotected through a sucrose gradient. A 2.0-cm segment of spinal cord centered at the injury area was sectioned at 20-µm thickness and thaw-mounted onto Superfrost Plus slides (Fisher Scientific) by placing serially on sequential sets of ten slides, each set representing a 200 µm length of spinal cord. A representative slide from each set was stained with eriochrome cyanine (ECRC) for myelinated white matter (blue). The lesion epicenter was identified as the section with the least amount of spared white matter [32].

### Immunohistochemistry

Immunohistochemistry was performed on spinal cord sections at 2 mm rostral and caudal to the injury epicenter as indicated in figures legends [31]. Water-bath antigen demasking was performed in 0.01 M sodium citrate solution, pH 9.0, for 30 min at 80°C for all antigens [33]. Sections were fixed with 10% buffered formalin after pre-warm at room temperature and blocked for 1 h with 10% normal goat serum in PBS +0.3% Triton X-100. For double and triple staining, the primary antibodies were mixed at optimal dilutions and subsequently detected using mixture of appropriate secondary antibodies. Cell nuclei were labeled with bis-benzimide solution (Hoechst 33258 dye, 5 µg/ml in PBS, Sigma). Finally, slides were washed and mounted with an anti-fading medium (Hydromount; National Diagnostics). Immunofluorescence was visualized by tile scan using a Leica TCS SP5 II Tunable Spectral Confocal microscope (Leica Microsystems Inc.). All immunohistological staining experiments were carried out with appropriate positive control tissue as well as secondary-only negative controls.

### Terminal deoxynucleotidyl transferase dUTP Nick-End Labeling [TUNEL]

To examine apoptotic DNA degradation *in vivo*, TUNEL analysis was performed on 20 µm sections from rats at 24 hours post-SCI using an ApopTag® Fluorescein/Red detection kit (Millipore) as described in the manufacturer's instructions. Standard fluorescent immunohistochemistry was employed with neuronal marker mouse anti-NeuN antibody to identify apoptotic neurons [2]. One µg/ml DAPI (Sigma) was used for counter staining.

### The human neuroblastoma SH-SY5Y cells, primary neuronal culture and transfections

The human neuroblastoma SH-SY5Y cells were seeded 60-mm Petri dishes and maintained in Dulbecco's Modified Eagle Medium (DMEM) supplemented with 10% fetal calf serum, 100 U/mL penicillin, and 100 U/mL streptomycin in a humid atmosphere of 5% CO2 and 95% air at 37°C. Cells at 70% confluence were transfected with 27 mer siRNA duplexes for human E2F1 (ID 1869) and trilencer-27 universal scrambled negative control siRNA duplex. Transfection of cells was performed by using Lipofectamine™ RNAiMAX (Invitrogen) according to manufacturer's protocol.

Cortical neurons were prepared from newborn Sprague-Dawley rats at postnatal day 0–1 as described [34]. Briefly, after dissection of pup brain, cortices were cultured in Basal Medium Eagle (BME) supplemented with 10% heat-inactivated bovine calf serum (Hyclone), 35 mM glucose, 1 mM L-glutamine, 100 U/mL of penicillin and 0.1 mg/ml streptomycin. Cytosine arabinoside (2.5 µM) was added to cultures on day 2 to inhibit the proliferation of non-neuronal cells. Cells were used for experiments on 4–7 days *in vitro* unless indicated otherwise. Transient transfections were performed on day 4 using the Lipofectamine 2000 reagent (Invitrogen) as described previously (Hetman et al., 2002). For E2F1/CDK1 protein expression analysis, transfection of neurons was performed by using rat neuron nucleofector® Kit (Lonza) according to manufacturer's protocol. The scrambled vector was used as a control. Pre-designed HusH-29 shRNA Sequences used for E2F1 and CDK1 knockdown experiments were as follows: E2F1 shRNA1 GATCACCTGATGCACATCTGTACCACTCA and shRNA 2 GAGCAAGAAGCTGTATTGCCTCGAATAGG; CDK1 shRNA1 GAGCGTTTGGAATACCGATAAGAGTGTAC and CDK1 shRNA2 GCCAGTTCATGGATTCTTCGCTCGTTAAG.

### Induction of neuronal apoptosis and immunofluorescence

Neuronal apoptosis was induced by trophic deprivation (TD) or camptothecin (10 µM). For TD neurons were placed in serum free media containing 10 µM MK801 as described before [35]. Apoptosis was identified by morphology of the cell nuclei, which were stained with Hoechst 33258. Condensed or fragmented nuclei were scored as apoptotic. In transfected cells, same criteria were applied to ß-galactozidase-positive cells only. At least 200 cells were evaluated for each condition in each independent experiment. For *in vitro* studies roscovitine was dissolved in 100% dimethyl sulfoxide (DMSO) and CR8 in sterile water. In experiments with cultured neurons these drugs were added at onset of trophic withdrawal.

For immunoflourescent analysis cells were fixed for 10 min at room temperature and washed three times in PBS, permeabilized with NP40 and processed for immunocytochemical staining using anti-β-galactosidase antibody as described elsewhere. Immunostaining by Alexaflour 594 goat antibodies against rabbit immunoglobulin (Molecular Probes) was used to detect transfected cells.

### NeuN^+^ cells counts by unbiased stereology

Transverse sections of intact and injured rat spinal cord at 35 days after injury were investigated using unbiased stereological quantification of NeuN-immunoreactive cells visualized with a DAB. Cresyl violet counterstaining was used to visualize nuclei. Unbiased stereology was used by the optical fractionation method with the aid of StereoInvestigator Software (MBF Biosciences). Sections spaced 1 mm apart from 5 mm caudal to 5 mm rostral to the injury epicenter were included for counting. A sampling grid composed of 150 µm by 150 µm squares in the gray matter was laid over each section. Cells were counted in a 50 µm by 50 µm counting frame within each square of the counting grid with a height of 10 mm and a guard zone of 4 mm from the top of the section. NeuN^+^ cells were counted throughout the entire section.

### Statistical Analysis

All the data were expressed as mean ± standard errors of the mean (SEM). The expressions of various proteins (% of sham) were analyzed by using Kruskal-Wallis one-way ANOVA based on ranks, followed by Dunnett's or Tukey's post-hoc test (Sigma Stat Program, Version 3.5, Systat Software). All other statistical tests were performed using the GraphPad Prism Program, Version 3.02 for Windows (GraphPad Software). A p<0.05 was considered statistically significant.

## Results

### Spinal cord injury causes upregulation of E2F1/CDK1 signaling pathway

**Figure 1:**
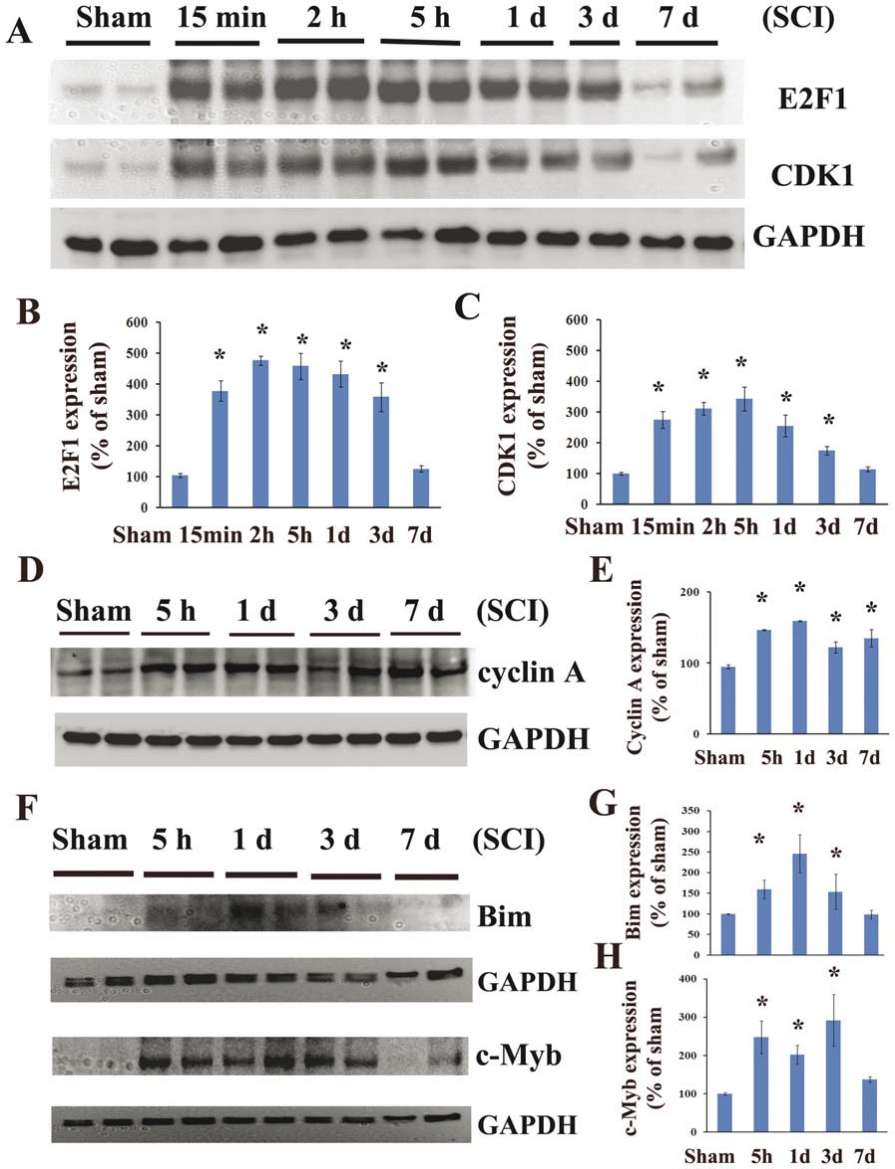
Spinal cord injury induces upregulation of expression of E2F1 and its downstream targets. Analysis of expression of E2F1 and its transcriptional target genes in intact and injured spinal cord were performed by western blotting. **A**. 5-mm-long segment centered at the injury epicenter was dissected and homogenized in RIPA buffer. Equal amounts of protein were electrophoretically separated on NuPAGE Novex Bis-Tris gradient gels, transferred to nitrocellulose membranes, and blotted with antibodies to E2F1 and CDK1. GAPDH signal served as a loading control. **B–C**. The signal quantifications for E2F1 (B) and CDK1 (C) using Gel-Pro Analyzer software are displayed. E2F1 and CDK1 expression level was upregulated as early as 15 min and sustained until 3 days after injury. **D–E**. Cyclin A expression was increased at all time points. **F**. Bim and c-Myb, downstream targets of E2F1 were upregulated as early as 5 h post-injury and sustained until day 3 after SCI. **G–H**. Quantification of respective western blots in panel D. N = 4 rats/time point. *P<0.05 vs sham group.

**Figure 2:**
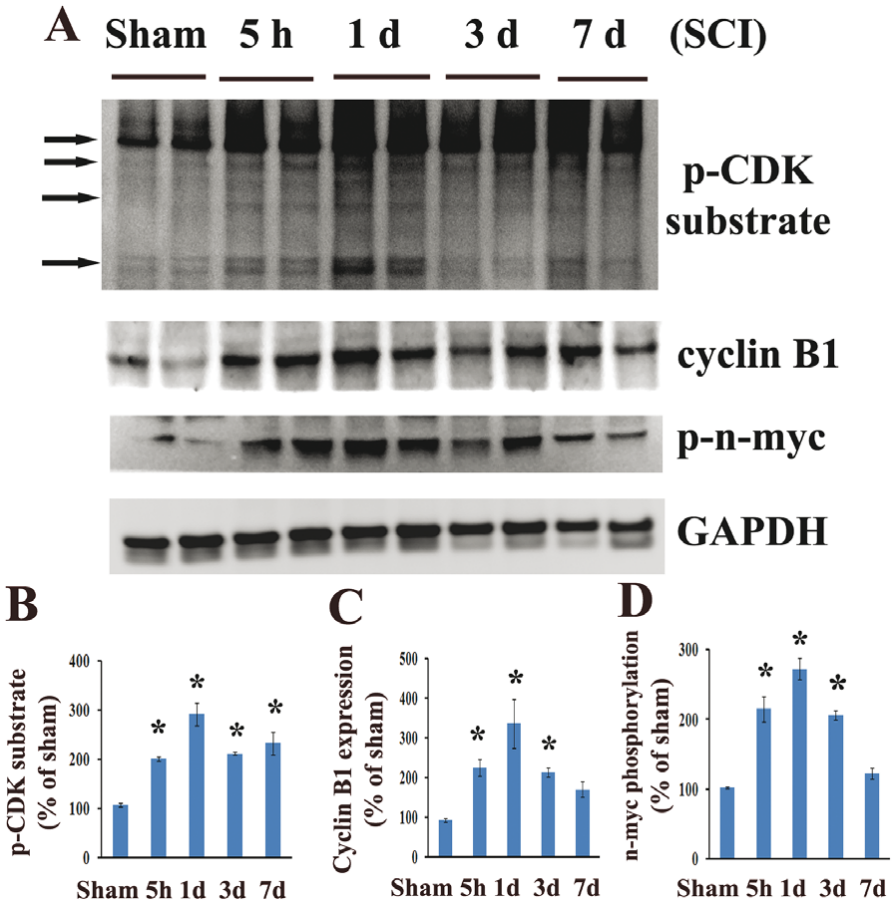
CDK1 activity is increased after spinal cord injury. Western blotting analysis of common CDK substrates, CDK1 co-activator cyclin B1 and phosphorylations of specific CDK1 substrate (Ser54)-n-myc was performed in homogenates obtained from intact and injured spinal cord. **A**. Cyclin B1 expression was upregulated at all time points tested. Phosphorylation (Ser54) of n-myc and phospho-CDK substrate motif signal levels were increased from 5 h to day 7. **B–D**. Quantification of respective western blots in panel A. n = 4 rats/time point. *P<0.05 vs sham group.

In order to explore the E2F1/CDK1 pathway following SCI we first performed western blotting analysis of the tissue obtained from the epicenter of the injured rat spinal cord. There was a significant increase of E2F1 protein levels, beginning as early as 15 min after injury, peaking at 2 h and continuing until day 3 after injury (Figure 1 A and B, 5-fold increase compared to control, p<0.05). CDK1 showed a similar pattern of up-regulation, but peaked at slightly later at 5 h after injury (Figure 1A and C, 3-fold). Cyclin A showed a similar, albeit more modest pattern of up-regulation (Figure 1 D and E, 1.5-fold of control). Further, Bim and c-Myb, downstream targets of E2F1 were upregulated as early as 5 h post-injury and sustained until day 3 after SCI (Figure 1 F–H, 1.5–3 fold). To determine if SCI induces cyclin dependent kinase activation we performed western blotting analysis of injured spinal cord samples using an antibody detecting the motif of the phosphorylated-(Ser) in CDK substrates (pCDK substrate, Figure 2A). We observed a significant increase in phospho-(Ser) CDKs substrates levels from 5 h to day 7 after injury (Figure 2B, 2.8-fold of control at day 1). Furthermore, our data suggested CDK1 activation following SCI based on elevation of CDK1 co-activator, cyclin B1 (Figure 2C, 3-fold of control at day 1, p<0.05) and increase in CDK1-specific phosphorylation site at Ser (54) of n-myc (Figure 2D, 2.7-fold of control at day 1, p<0.05).

**Figure 3:**
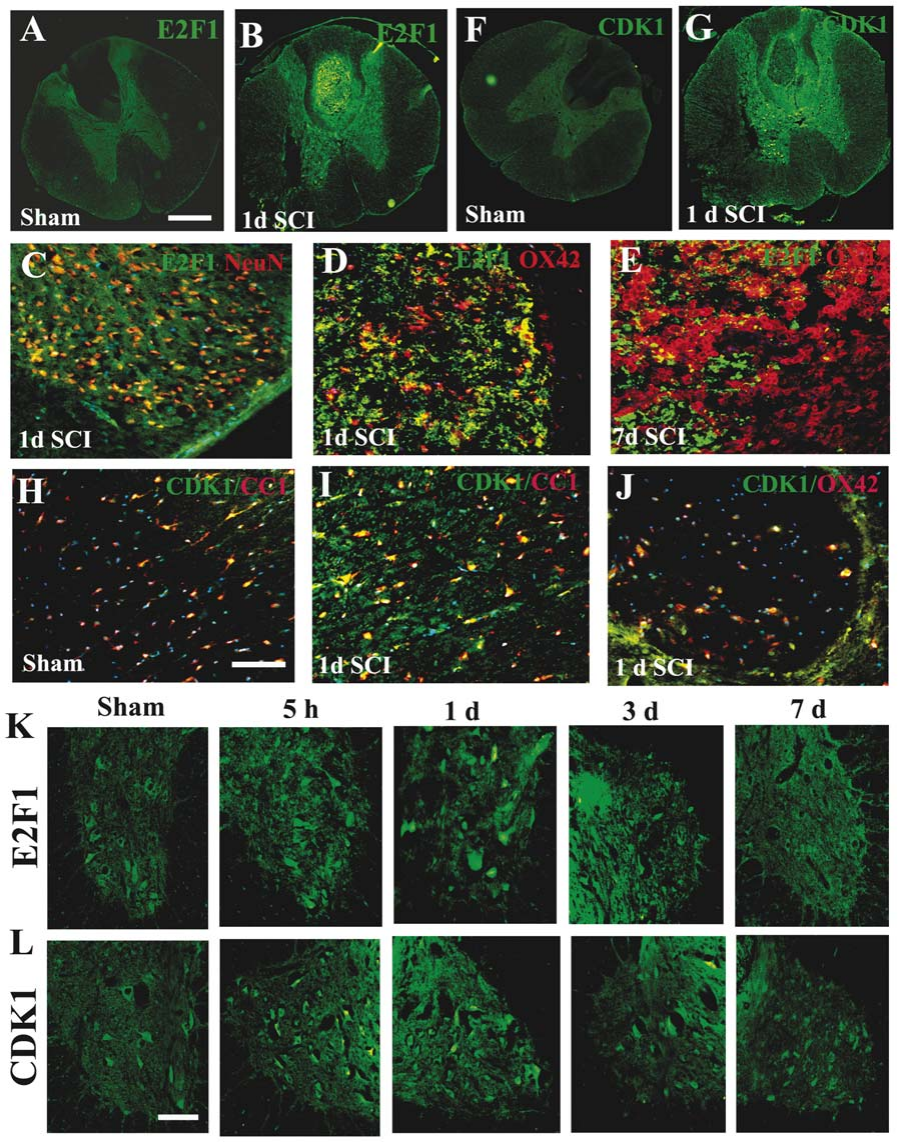
The temporal profile and cell specificity of E2F1 and CDK1 expression after SCI. **A–B**. Coronal section in intact spinal cord (A) showed that E2F1 is relatively weak and detected mainly in neurons of the gray matter. At 24 h after SCI, E2F1 immunoreactivity was upregulated not only in gray matter but also in lesion area (B). C. E2F1^+^ cells were also co-labelled with NeuN in the dorsal horn of the gray matter at 1 day after SCI. D–E. Only a small subset of E2F1^+^ cells in the lesion area were positive for OX42 at 24 h (D) and 7 d (E) after SCI. F–G. In the intact spinal cord (F), CDK1 immunoreactivity is relatively weak and detected mainly in motor neurons in the ventral horn and CC1^+^ oligodendrocytes. At 24 h after SCI (G), CDK1 immunoreactivity was upregulated not only in the ventral horn but also in the spared white matter, colocalized with CC1^+^ oligodendrocytes. CDK1^+^ cells also appeared in the lesion area. **H–I**. CDK1 was expressed by CC1^+^ oligodendrocytes in the white matter in the intact spinal cord (H) and at 1 day after SCI. **J**. Only a small subset of CDK1^+^ cells in the lesion area were positive for OX42 at 24 h after SCI. **K**. Coronal section in intact spinal cord (a) shows that E2F1 was expressed in the motor neurons in the ventral horn. Immunoreactivity of E2F1 (b–d) was increased at 5 h, and 1–3 days post injury, and highly expressed by motor neurons. L. In intact spinal cord (a), CDK1/NeuN was detected in the motor neurons in the ventral horn. At 5 h after injury, immunoreactivity of CDK1 (b) was increased and sustained until 3 days post injury (c–d), and highly expressed by motor neurons. All images were taken at 2 mm rostral to epicenter. Scale bar = 500 µm for A–B, F–G. Scale bar = 100 µm for C–E, H–J, K–L.

Next, we performed a comprehensive immunohistochemistry study to examine the temporal profile of E2F1/CDK1 expression and cell specificity after SCI. In the intact spinal cord, E2F1 immunoreactivity was weakly detected in neurons across the gray matter (Figure 3 A & Ka). E2F1 neuronal immunoreactivity was upregulated at 5 h after injury (Figure 3Kb) and sustained until 3 days post injury (Figure 3 B, C, Kb–d). At 24 h after SCI, E2F1 immunoreactivity was upregulated not only in gray matter but also in lesion area (Figure 3 B, D, E), this patter persisted at 7 days (Figure 3 E). Only a small subset of these cells was positive for OX42, suggesting that the majority E2F1^+^ cells in the lesion area is infiltrating circulating cells (Figure 3 D & E). CDK1 immunoreactivity is relatively weak in the intact spinal cord and detected mainly in motor neurons in the ventral horn and CC1^+^ oligodendrocytes (Figure 3 F& H). CDK1 immunoreactivity in motor neurons in the ventral horn was upregulated at 5 h after injury (Figure 3 Lb) and sustained until 3 days post injury (Figure 3 G & Lb–d). At 24 h after SCI, CDK1 immunoreactivity was upregulated not only in the ventral horn but also in the spared white matter, colocalized with CC1^+^ oligodendrocytes (Figure 3 I). CDK1^+^ cells also appeared in the lesion area although not colocalized with OX42 suggesting that they may represent infiltrating circulating cells (Figure 3 G & J). At 7 days after SCI, double-labelling of CDK1^+^/NeuN^+^ cells was rarely seen (Figure 3 Le), however, CDK1^+^/CC1^+^ cells were continuously presented in spared white matter (data not shown).

### E2F1 gene silencing down-regulates endogenous CDK1 expression *in vitro*

**Figure 4:**
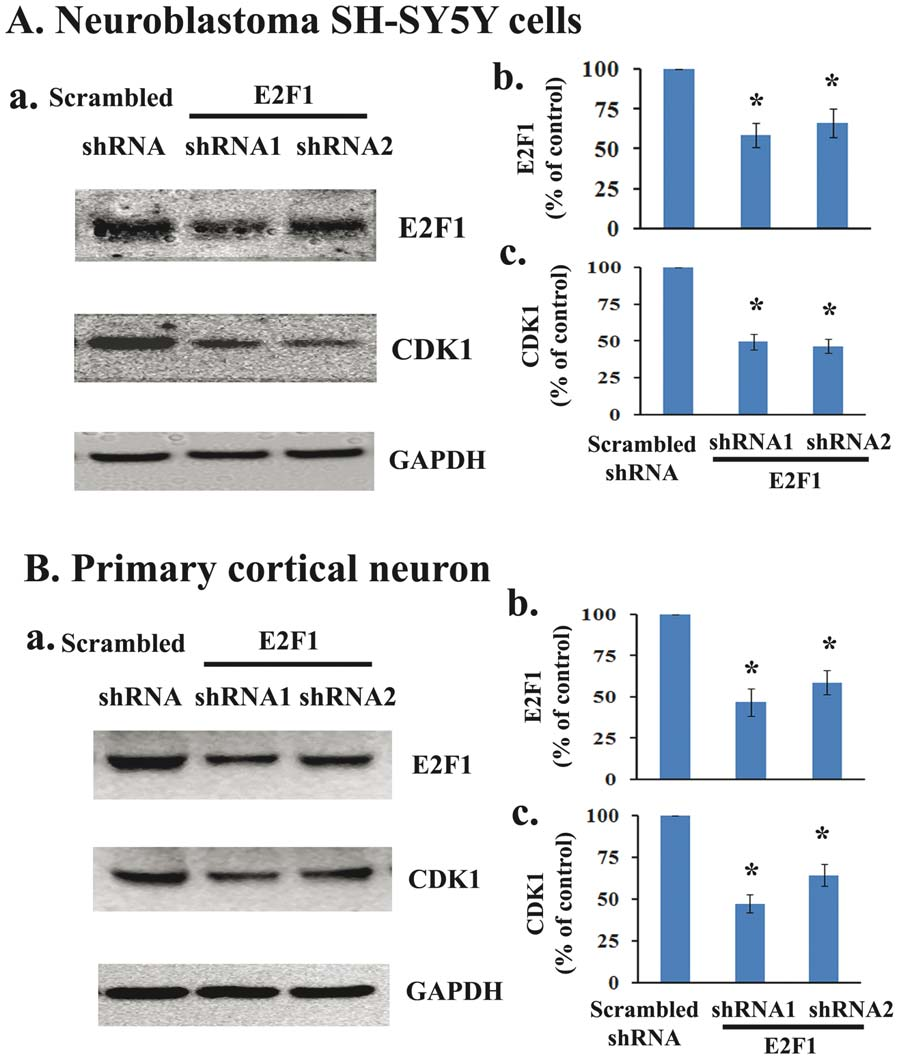
E2F1 gene silencing down-regulates endogenous CDK1 expression *in vitro*. **A**. 27 mer siRNA duplexes for human E2F1 or trilencer-27 universal scrambled negative control siRNA duplex was transfected in the human neuroblastoma SH-SY5Y cells. Two days after transfection, the cells were harvested and subjected to western blotting using mouse monoclonal antibodies to E2F1 and CDK1. Transfection with shRNA against E2F1 resulted in reduction of E2F1 expression (58% to 66% of control), accompanied by 50% of reduction of CDK1 expression. **B**. Primary rat cerebral cortical neurons were transfected with shRNA against rat E2F1. E2F1 protein expression was robust reduced to 47% or 59% for shRNAs 1 and 2 respectively, and E2F1 knockdown resulted in reduction of CDK1 expression from 47% to 64% for shRNAs 1 and 2 respectively. N = 4 dishes from 3 independent culture. *P<0.05 vs sham group.

We next examined whether E2F1 has the ability to regulate the expression of the endogenous CDK1 *in vitro*. First, we transfected the human neuroblastoma SH-SY5Y cells with 27 mer siRNA duplexes for human E2F1 or trilencer-27 universal scrambled negative control siRNA duplex. Two days after transfection, the cells were harvested and subjected to western blotting using mouse monoclonal antibodies to E2F1 and CDK1. Transfection with shRNA against E2F1 resulted in a significant reduction of E2F1 expression (58% to 66% of control), accompanied by a significant 50% of reduction of CDK1 expression (Figure 4 A). Further, primary rat cortical neurons were transfected with shRNA against rat E2F1. We found that E2F1 protein expression was significantly reduced to 47% or 59% for shRNAs 1 and 2 respectively, and E2F1 knockdown resulted in a significant reduction of CDK1 expression from 47% to 64% for shRNAs 1 and 2 respectively (Figure 4 B). These results demonstrate that decreasing E2F1 expression by shRNAs down-regulates CDK1 expression, consistent with reports by others (Konishi Y and Bonni A, 2003; Yuan et al., 2008).

### Elevated E2F1/CDK1 expression is associated with apoptotic markers in neurons following spinal cord injury

**Figure 5:**
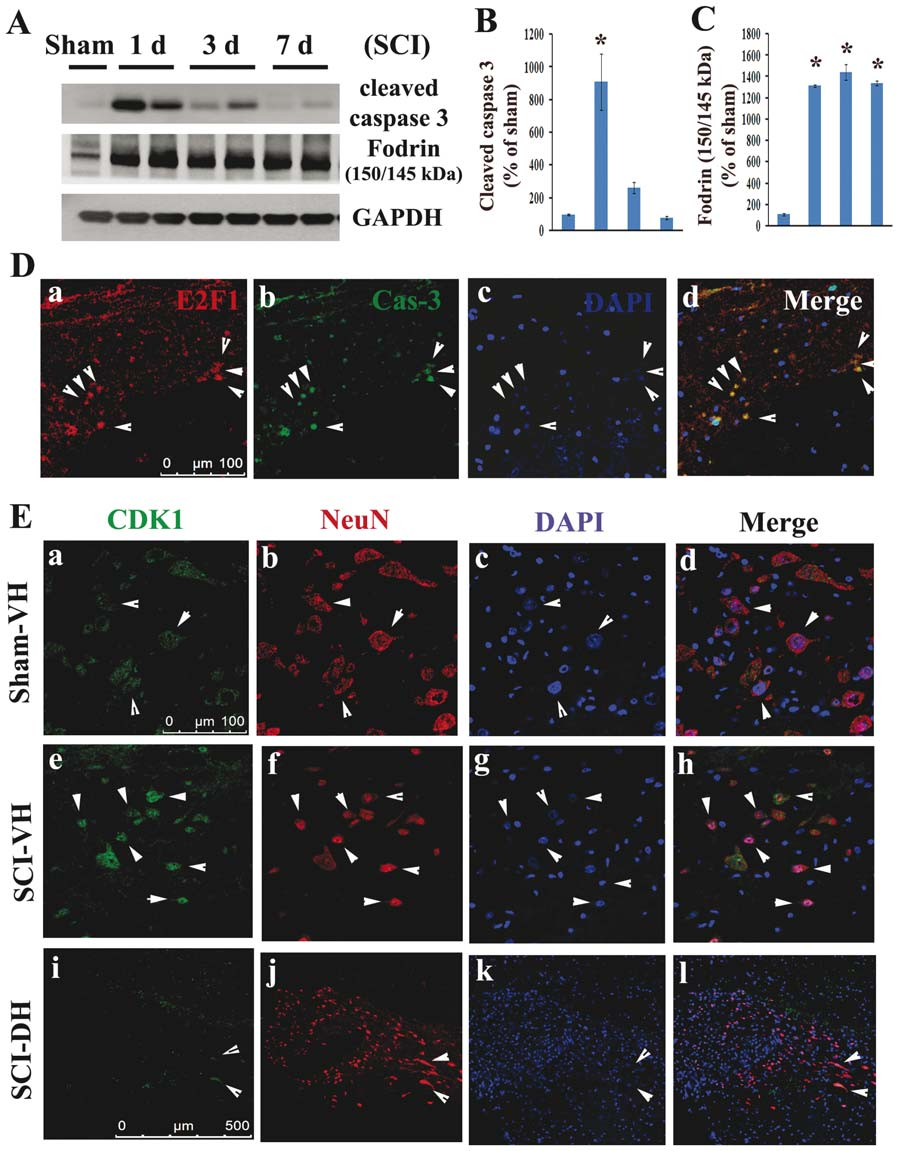
Colocalization of E2F1/CDK1 upregulation with neuronal apoptosis in injured spinal cord. **A–C**. Western blot analysis shows **a significant increase** in biochemical markers of apoptosis, active caspase-3 signal, as well as 145/150 kDa cleavage product of α-fodrin after SCI. N = 4 rats/time points. *p<0.05 vs sham group. **D**. E2F1^+^ cells were co-label with cleaved caspase 3 (yellow, arrow heads) in the gray matter at 2 mm rostral to the epicenter at 1 day after SCI. Scale bar = 100 µm. **E**. Coronal section in intact spinal cord (top panel) shows that CDK1 was expressed in the motor neurons in the ventral horn (VH). At 1 day after injury, immunoreactivity of CDK1 (middle panel, green) was increased, and highly expressed by apoptotic motor neurons (red), as shown at 2 mm rostral to epicenter. CDK1 was rarely expressed by inter-neurons in the dorsal horn (DH) after SCI (bottom panel). Scale bar = 100 µm for D(a–h) and 500 µm for D(i–l).

In spinal cord injury samples we detected significant increases in biochemical markers of apoptosis, including active caspase-3 as well as the 145/150 kDa cleavage product of α-fodrin. Activation of caspase-3 peaked at day 1 (Figure 5A–B, 9-fold of control p<0.05), while α-fodrin cleavage product was significantly elevated at day 1 and remained elevated for at least 7 days post-injury (Figure 5A& C, 13-fold increase at day 1, p<0.05). Immunohistochemistry showed that many of the apoptotic cells (cleaved caspase-3-positive) in the gray matter at 1 day after SCI were co-labeled with markers of cell cycle activation (E2F1-positive) (Figure 5Da–d). SCI also induced up-regulation of CDK1 in neurons that show morphological features of apoptosis. Coronal sections in sham-injured spinal cord showed that CDK1 was expressed in the motor neurons in the ventral horn (VH) (Figure 5Ea–d). The nuclei of these NeuN-positive cells appeared large and uniformly stained with DAPI. In contrast, at 1 day after injury, immunoreactivity of CDK1 was increased in motor neurons which also displayed condensed, fragmented or shrink nuclei (Figure 5Ef–h). CDK1 expression was rarely observed after SCI in dorsal horn interneurons (Figure 5Ei–l).

### E2F1 and CDK1 promote apoptosis in primary cortical neurons

**Figure 6:**
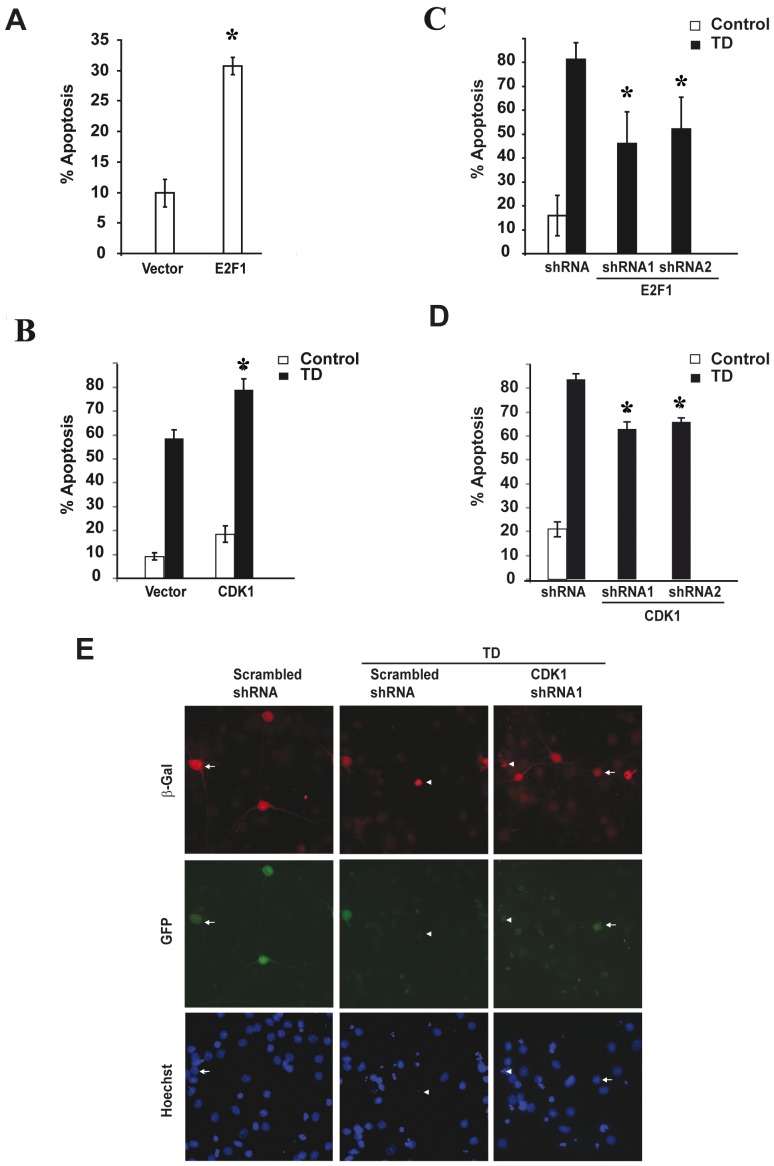
E2F1/CDK1 expression is necessary for trophic deprivation-induced neuronal apoptosis. Rat cortical neurons were co-transfected with expression plasmids for ß-galactosidase with, either empty vector or vector expressing E2F1 or CDK1 (A and B). Similarly, ß-galactosidase plasmid was co-transfected along with scrambled, E2F1 or CDK1 shRNAs (C, D and E) and the extent of apoptosis was examined 48 h after transfection, or after an additional 24 h of trophic deprivation (TD) induction post 48 h transfection. **A**. Neurons transfected with E2F1 vector (0.6 µg DNA/0.5×10^6^ neurons) increased basal apoptosis as compared to empty vector. **B**. Neurons transfected with CDK1 (0.8 µg DNA/0.5×10^6^ neurons) vector enhanced TD-induced apoptosis as compared to empty vector transfected cells. **C–D**. A quantitative assessment of the percentage of nuclei featuring chromatin condensation demonstrates a significant attenuation of TD-induced apoptosis in neurons transfected with two different shRNAs targeting either E2F1 or CDK1. **E**. Attenuation of TD-induced chromatin condensation in neurons transfected by CDK1 shRNA1 is shown. Representative photomicrographs of the shRNA transfected neurons, which also co-express GFP protein from the separate promoter are shown. In all experiments ß-galactosidase was co-transfected at 0.01 µg DNA/0.5×10^6^ neurons to visualize transfected neurons at later apoptotic stage. Neurons with either normal diffuse chromatin morphology or apoptotic condensation of nuclei (after Hoechst 33258 chromatin staining) are indicated by arrowheads or arrows, respectively. Statistical analysis was performed by Kruskal-Wallis one-way ANOVA on ranks, followed by post hoc adjustments using Dunnett's test. For A ^*^p<0.001, vs. vector; For C ^*^p<0.01, vs. TD vector; For D ^*^p<0.05, vs. TD vector; Error bars are ± S.E.M. from three independent experiments.

To directly explore the consequences of overexpression of E2F1 or CDK1 in neurons we transfected primary cortical neurons with expression vectors encoding E2F1, CDK1 or control vector. E2F1 overexpression was sufficient to induce neuronal apoptosis, increasing basal neuronal death from about 10% to 28% in 48 hours after transfection (Figure 6A, p<0.05, vs. TD vector). Although overexpression of the CDK1 construct was not sufficient to significantly elevate basal neuronal death, over-expression of this protein enhanced Trophic Deprivation (TD) induced apoptosis by 34% relative to neurons transfected with control vector. The percentage of apoptotic cells reached 79% in the CDK1 sample from 59% in the control vector sample (Figure 6B, p<0.05, vs. TD vector). There was neither induction nor enhancement of apoptosis when cyclin A and B1 was expressed or co-expressed along with CDK1 (data not shown).

To further investigate the role of E2F1/CDK1 pathway in neuronal apoptosis, we examined whether decreasing CDK1 or E2F1 expression by shRNAs could attenuate trophic withdrawal-induced apoptosis. Neurons were transfected with two different plasmids, each expressing green fluorescent protein along with a distinct shRNA against rat E2F1 or CDK1. 48 hours later apoptosis was induced by TD. After an additional 24 hours we quantified the percentage of apoptotic cells, identified by nuclei featuring chromatin condensation. E2F1 knockdown resulted in reduction of apoptosis from 81% to 46% or to 53% for shRNAs 1 and 2 respectively (Figure 6C, p<0.001 and p<0.01 for shRNA1 or shRNA2 vs. control shRNA, respectively). Both shRNA constructs against CDK1 resulted in greater than 20% decrease of TD-induced neuronal apoptosis relative to scrambled control. shRNA1 reduced the percentage of apoptotic neurons to 63% and shRNA2 to 66% percent as compared to 84% in scrambled control (Figure 6D, p<0.05, vs. TD control shRNA). Within beta-galactosidase positive population, CDK1 shRNA1 (indicated by GFP) expressing cells displayed a higher proportion of healthy nuclei as compared to scrambled shRNA expressing neurons (Figure 6E). Hence, reducing CDK1 or E2F1 proteins levels provides a significant attenuation of neuronal apoptosis in our paradigm suggesting that CDK1 and E2F1 are necessary for TD-induced neuronal cell death.

### Pharmacological inhibition of CDK1 block apoptosis in primary cortical neurons

**Figure 7:**
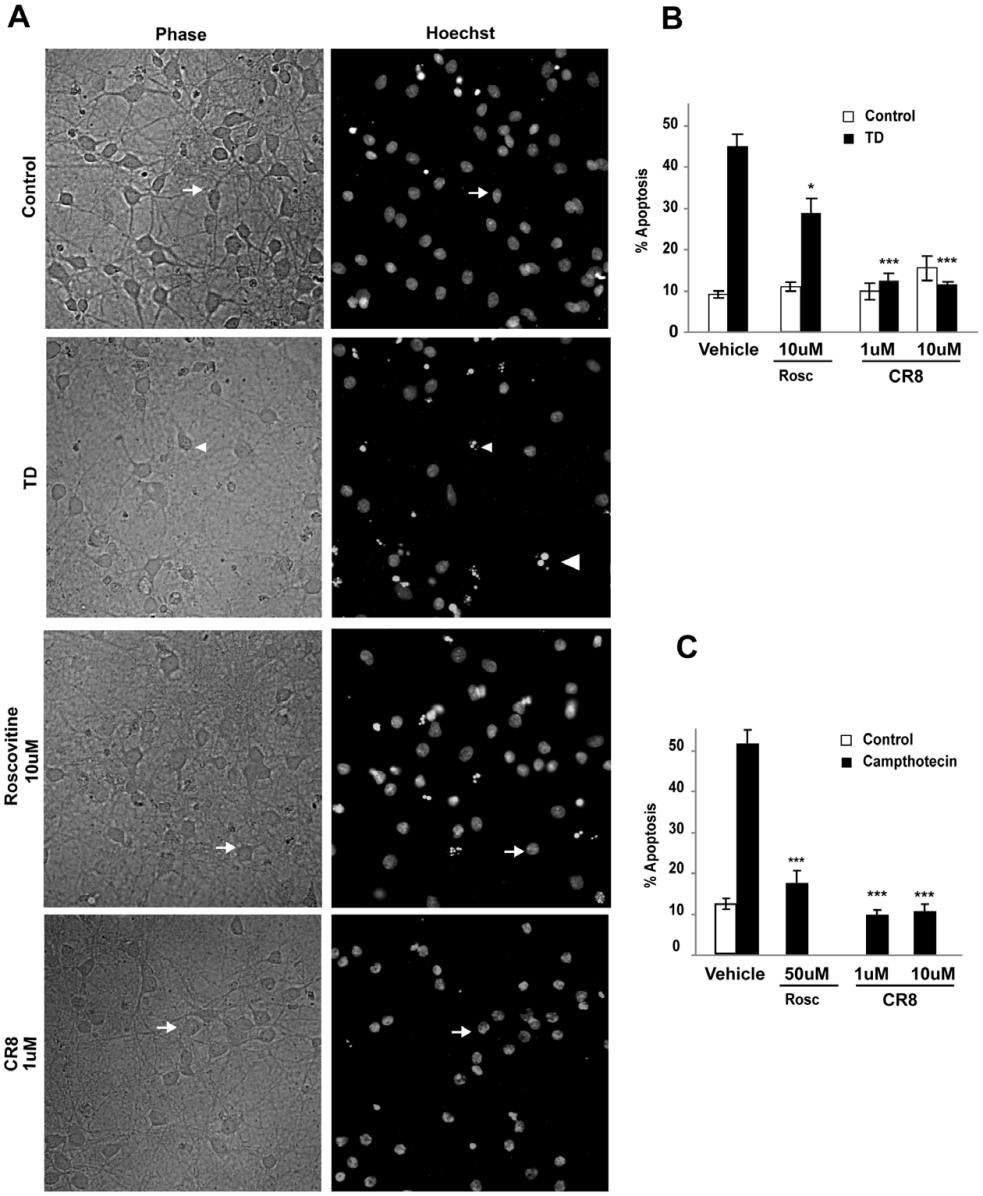
Pharmacological inhibition of CDK1 blocks neuronal apoptosis. Cortical neurons were pre-treated with Roscovitine or CR8 (CDK1 inhibitors) or vehicle and then exposed to TD-or campthotecin induced apoptosis. **A**. Representative photomicrographs of control and trophic deprived neurons treated with the indicated concentrations Roscovitine and CR8 are shown. Upper row presents phase contrast images (Healthy neurons are indicated by larger cell bodies and abundant processes; Apoptotic neurons display shrunken cell bodies and sparse or lost processes). Lower row shows chromatin staining with Hoechst 33258. Arrows and arrowheads indicate surviving and apoptotic neurons, respectively suggesting an attenuation of TD-induced neuronal death in neurons pre-treated with Roscovinine or CR8. **B**. A quantitative assessment of the percentage of nuclei featuring chromatin condensation demonstrates a significant attenuation of TD-induced apoptosis in neurons pre-treated with Roscovitine (10 µM; ^*^p<0.05, vs. TD vehicle) whereas CR8 at concentrations as low as 1 µM (^***^p<0.001, vs. TD vehicle) almost completely blocked development of apoptotic features in neuronal nuclei. **C**. Significant attenuation of campthotecin-induced apoptosis in neurons pre-treated with Roscovitine (50 µM; ^*^p<0.001, vs. vehicle) and CR8 at concentrations as low as 1 µM (^***^p<0.001, vs. vehicle).

To confirm the role of CDK1 in neuronal apoptosis we used a model of pharmacological inhibition with two relatively selective CDK1 inhibitors, Roscovitine and CR8. Protection was indicated by decreased number of neurons with shrunken cell bodies and reduced percentage of nuclei displaying chromatin condensation. Roscovitine treatment at a concentration of 10 µM or 50 µM significantly attenuated, TD-induced (Figure 7A–B) or campthotecin-induced neuronal apoptosis (Figure 7C), respectively. Roscovitine (10 µM) reduced TD-induced apoptosis to 30% as compared to 42% in the vehicle-treated neurons (Figure 7A–B; p<0.05, vs. TD vehicle). In this model, CR8 conferred neuroprotection at concentrations as low as 1 µM, reducing apoptotic neurons to almost control level (Figure 7B; 12%, 11% apoptosis in neurons treated with 1 µM and 10 µM CR8 respectively; p<0.001, versus TD vehicle). In the model of campthotecine-induced neuronal apoptosis Roscovitine (50 µM) decreased apoptosis from 52% to 16%, while CR8 confirmed it's higher potency reducing apoptosis to 10% at concentrations as low as 1 µM (Figure 7C). These data support our hypothesis that inhibition of CDK1 signaling protects neurons from apoptosis.

### CR8 suppresses activation of E2F1/CDK1 pathway following SCI

**Figure 8:**
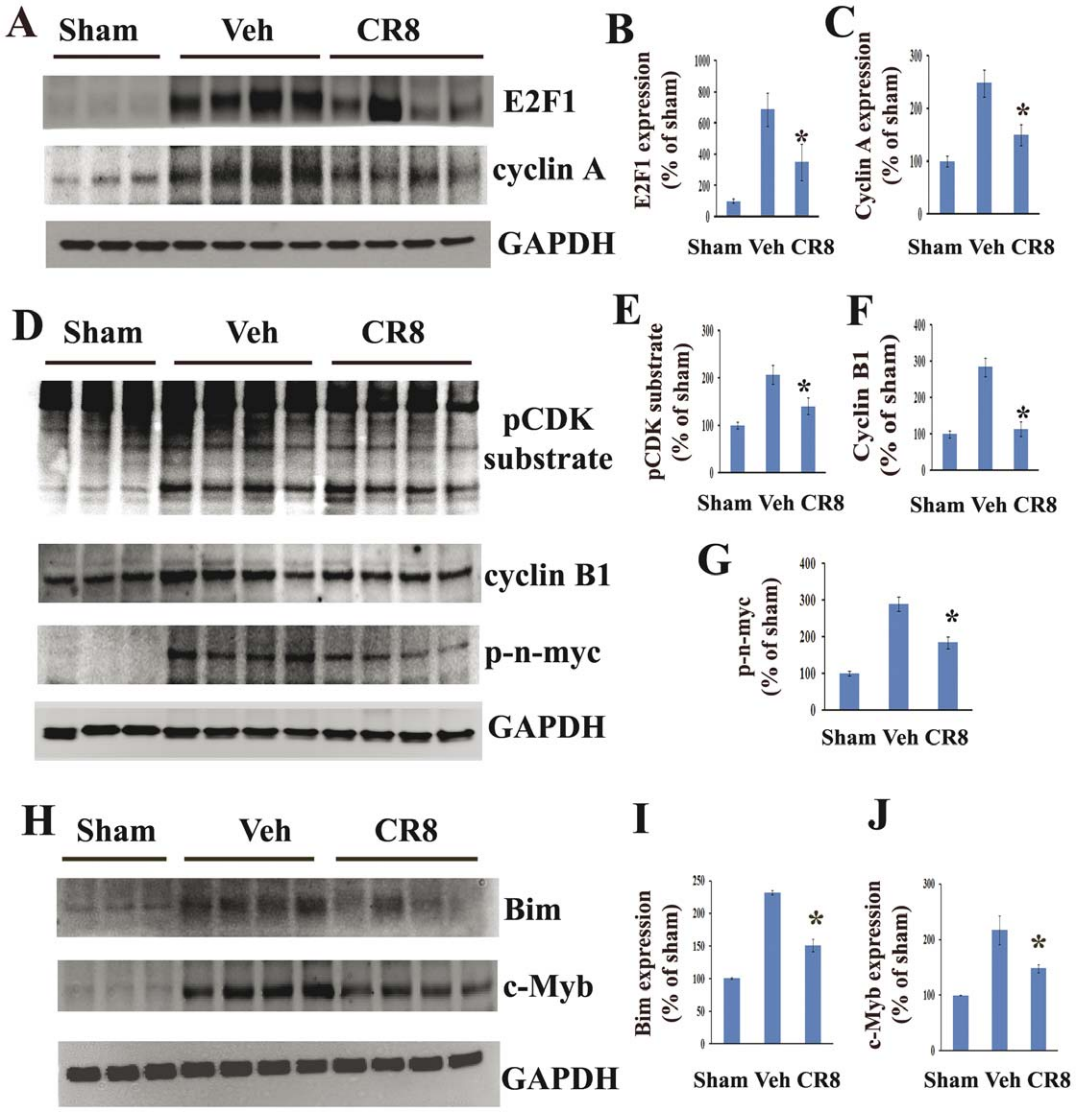
CR8 administration reduces SCI-induced activation of the E2F1/CDK1 signaling pathway. Samples were obtained from rats exposed to spinal cord injury and CR8 treatment (1 mg/kg intraperitoneal administration) and analyzed by western blotting. Equal protein loading is demonstrated by consistent GAPDH levels. **A**. CR8 attenuated SCI mediated increase in E2F1 and its target cyclin A expression. **B–C**. Quantification of respective western blots in panel A. **D**. CR8 reduced SCI induced increase in phospho-(Ser54)-n-myc, phosphorylated CDK substrates and expression of cyclin B1. **E–G**. Quantification of respective western blots in panel E. **H–J**. Administration of CR8 significantly reduced Bim and c-Myb expression at 24 h after SCI. H shows representative Western blots for Bim, c-Myb, and the loading control, GAPDH. I and J show quantitative analysis of Bim and c-Myb expression. N = 4. ^*^p<0.05 vs. vehicle group.

We investigated whether SCI-induced activation of E2F1/CDK1 pathway could be attenuated by systemic administration of CR8 *in vivo*. Rats were treated with CR8 or saline by ip injection at 5 min post injury and spinal cord tissue was collected at 24 h after SCI for Western blot analysis. At 24 h after SCI, E2F1 and cyclin A expression increased by approximately 7- or 2.5-fold, respectively as compared to sham-injured animals (Figure 8A–C), which is consistent with the observation in Figure 1. Notably, CR8 treatment significantly attenuated SCI-induced increase of both E2F1 and its target gene cyclin A expression (Figure 8A–C, p<0.05, vs. vehicle). Importantly, administration of CR8 significantly reduced downstream targets Bim and c-Myb expression at 24 h after SCI (Figure 8 H–J). We also observed significant inhibition of total CDK activity by CR8 treatment as indicated by decreased levels of phosphorylated-(Ser) in CDK substrates (1.5-fold reduction of vehicle, p<0.05, vs. vehicle) (Figure 8D, E). CR8 inhibited the CDK1 pathway as indicated by the attenuation of SCI-induced increase of CDK1 co-activator, cyclin B1 expression (Figure 8D, F, p<0.05, vs. vehicle). Finally, CR8 administration also attenuated SCI-induced CDK1 activation, as evidenced by inhibition in CDK1 substrate n-myc phosphorylation at Ser (54) (Figure 8D, G, 1.5-fold decrease of vehicle, p<0.05, vs. vehicle).

**Figure 9:**
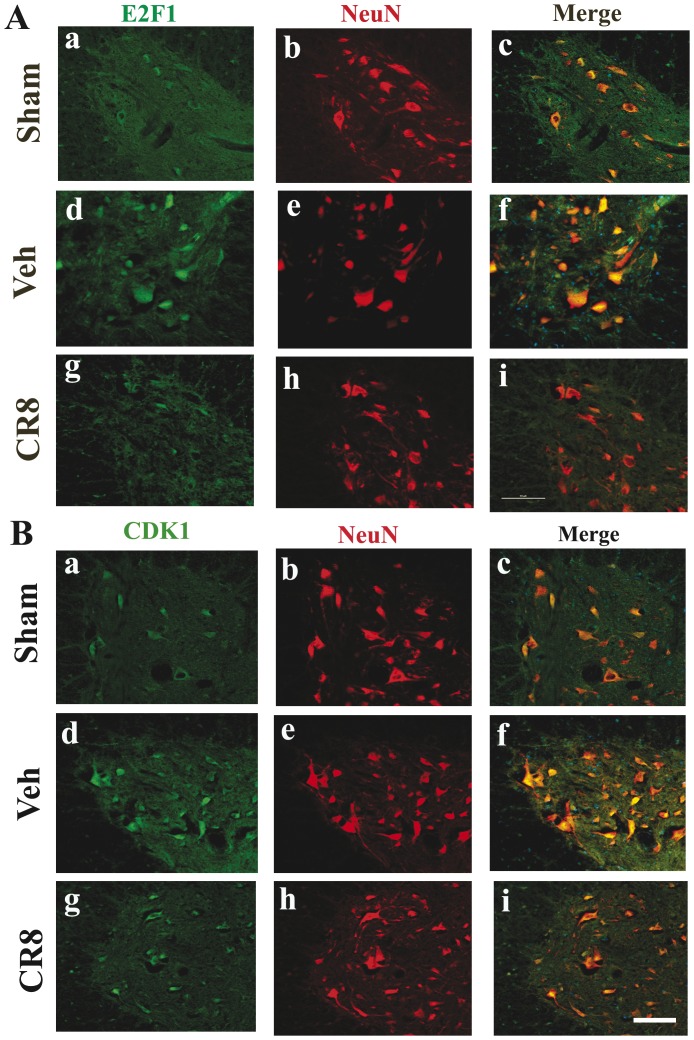
SCI-induced immunoreactivity of E2F1 and CDK1 was attenuated by CR8 treatment. **A**. Coronal section in intact spinal cord (a–c) shows that E2F1 was expressed in the motor neurons in the ventral horn. At 1 day after injury, immunoreactivity of E2F1 (d–f) was increased, and highly expressed by motor neurons. The upregulation of E2F1 was clearly attenuated by CR8 treatment (g–i). **B**. In intact spinal cord (a–c), CDK1/NeuN was detected in the motor neurons in the ventral horn. At 1 day after injury, immunoreactivity of CDK1 (d–f) was increased, and highly expressed by motor neurons. CDK1 upregulation was attenuated by CR8 treatment (g–i). All images were taken at 2 mm rostral to epicenter. Scale bar = 100 µm for C–F.

**Figure 10:**
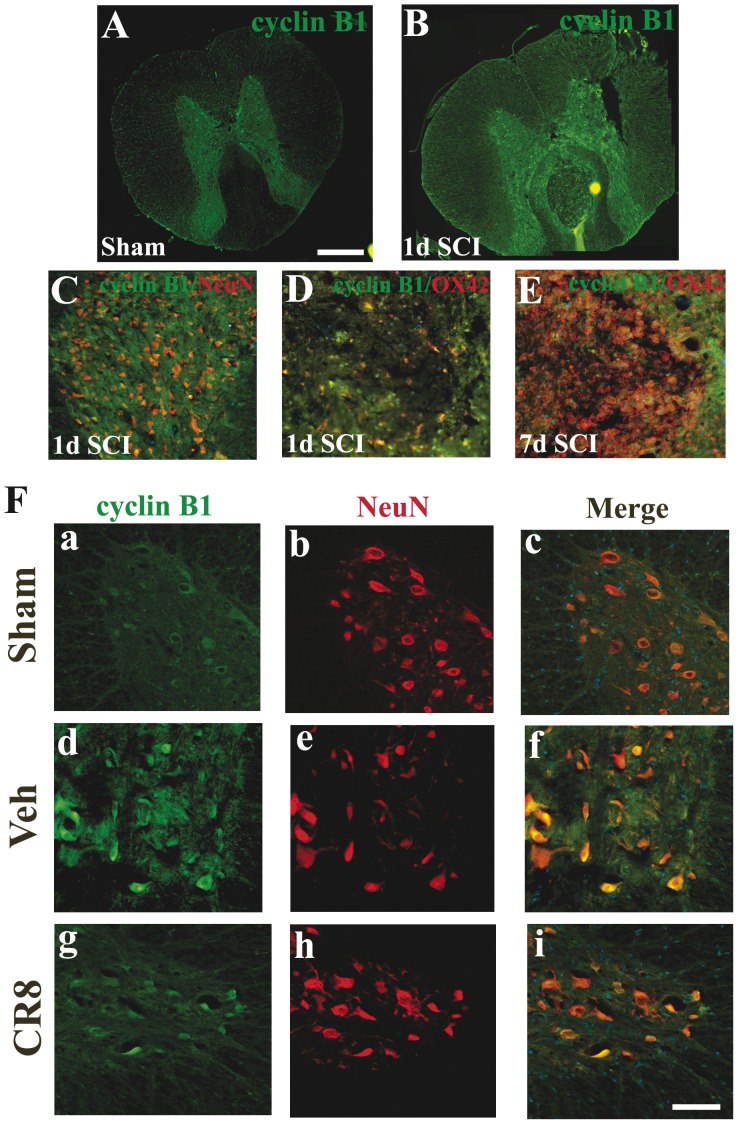
The temporal profile, distribution and the cellular localization of cyclin B1 after SCI. **A–B**. In the intact spinal cord (A), cyclin B1 immunoreactivity is relatively weak and detected mainly in neurons in the gray matter. At 24 h after SCI (B), cyclin B1 immunoreactivity was upregulated not only in the gray matter but also in the lesion area. **C**. Cyclin B1 was also expressed by neurons in the dorsal horn at 1 day after SCI. **D–E**. Most of cyclin B1^+^ cells is OX42 negative at 24 h (D) and 7 d (E) after SCI, suggesting they are infiltrating circulating cells. **F**. Coronal section in intact spinal cord (a–c) shows that cyclin B1 was expressed in the motor neurons in the ventral horn. At 1 day after injury, immunoreactivity of cyclin B1 (d–f) was increased, and highly expressed by motor neurons. Cyclin B1 upregulation was attenuated by CR8 treatment (g–i). All images were taken at 2 mm rostral to epicenter. Scale bar = 500 µm for A–B; Scale bar = 100 µm for C–F.

Double-label immunohistochemistry confirmed that immunoreactivity of E2F1/CDK1 was increased by neuron in the ventral horn at 1 day after SCI (Figure 9). Importantly, the upregulation of both CDK1 and E2F1 was clearly attenuated by CR8 treatment (Figure 9 A & B). In addition, we found that cyclin B1 immunoreactivity was upregulated as early as 5 h (data not shown) and reached a peak 1 day post injury predominantly in neurons in gray matter (Figure 10). At 24 h after SCI, cyclin B1 immunoreactivity was upregulated not only in the gray matter but also in the lesion area (Figure 10 B–E). As in the case of E2F1 and CDK1, most of these cells are OX42 negative suggesting they are infiltrating circulating cells (Figure 10 B, D–E). Moreover, cyclin B1 upregulation was attenuated by CR8 treatment (Figure 10 F). Altogether, these data indicate that intraperitoneal administration of 1 mg/kg CR8 significantly inhibits SCI-induced up-regulation of E2F1/CDK1 signaling pathway.

### CR8 reduces neuronal apoptosis after SCI

**Figure 11:**
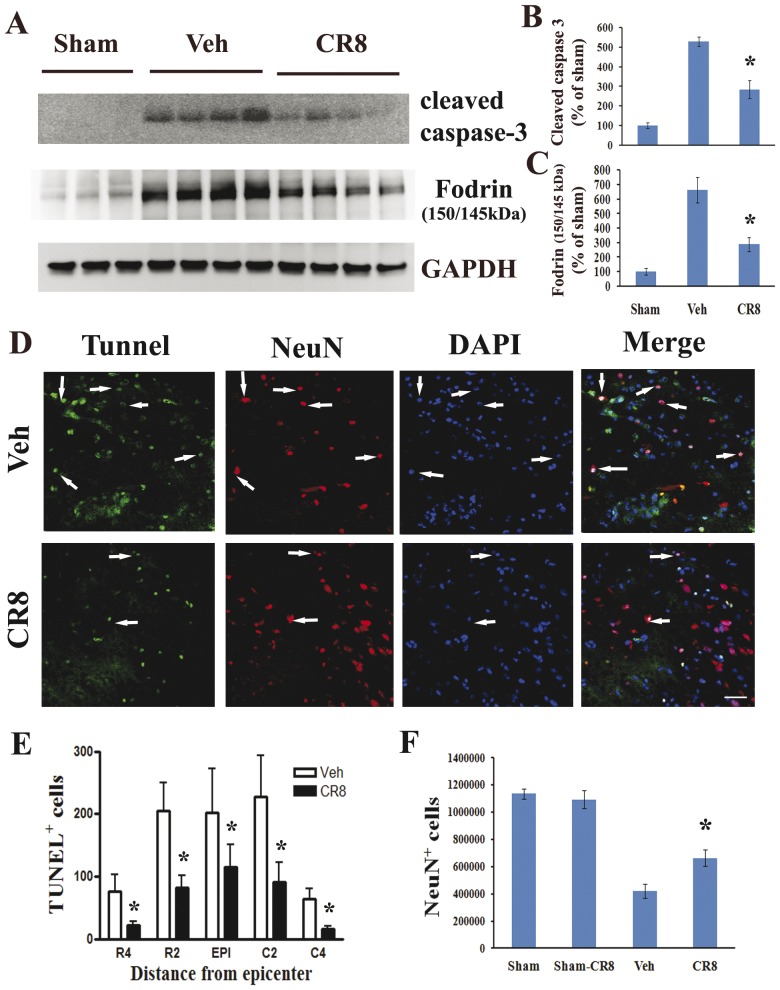
CR8 administration reduces SCI-induced neuronal death. **A**. CR8 attenuated caspase 3 and fodrin cleavage, the latter as indicated by reduction of the 145/150 kDa fragment immunoreactivity. **B–C**. Signal quantification using densitometry and normalization relative to GAPDH levels demonstrated that the observed changes are significant. The figures reflect representative western blots. N = 4. ^*^p<0.05 vs. sham. **D–E**. TUNEL analysis was performed on 20 µm sections from rats at 24 hours post-SCI using an ApopTag® Fluorescein/Red detection kit. TUNEL/NeuN staining revealed that CR8 effectively reduced neuronal death in the epicenter of the injury (p<0.05), as well as in rostral and caudal sections located in two consecutive 2 mm distance regions from the epicenter. Representative images in panel D were shown at 2 mm caudal to epicenter. Scale bar = 100 µm. **F**. Six to eight representative animals - were selected from each treatment group for quantification of neurons (NeuN^+^ cells). Unbiased stereology by optical fractionators method using StereoInvestigator Software (MBF Biosciences) indicates that CR8 administration significantly increased number of surviving neurons within 10 mm zone surrounding the injury epicenter (p<0.05, vs vehicle).

In order to determine the significance of CR8-mediated inhibition of cell cycle activation we tested the neuroprotective effects of CR8 in the *in vivo* model of contusion spinal cord injury. First, biochemical markers of apoptosis, such as active caspase-3, as well as cleaved fragments of α-fodrin were assessed in the injured spinal cord by Western blotting analysis. Active caspase-3 signal was increased approximately 7-fold by 24 h after SCI, while 145/150 kDa cleavage fragment of the α-fodrin was increased 6-fold, as compared to sham-injury (Figure 11 A–C). Notably, CR8 administration significantly reduced these markers of apoptosis. Cleaved caspase-3 signal decreased to 2-fold of vehicle (Figure 11 A–B, p<0.05 CR8 vs. Vehicle). 145/150 kDa cleavage fragment of the α-fodrin was significantly reduced to 3-fold of vehicle (Figure 11 A–C, p<0.05 CR8 vs. Vehicle).

Neuronal apoptosis was evaluated by immunohistochemical analysis of spinal cord sections using TUNEL/NeuN staining. Cell nuclei were indicated by chromatin staining using DAPI. Numerous TUNEL-positive cells were observed in the vehicle-treated spinal cord, in the epicenter, as well as 4 mm rostral or caudal to it at 24 h post injury (Figure 11 D). Most of these appeared in NeuN-positive cells in the gray matter indicative of neuronal apoptosis (Figure 11 D). CR8 significantly reduced TUNEL positive cell in the epicenter of the injury, as well as in rostral and caudal sections located in two consecutive 2 mm distance regions from the epicenter. In the epicenter, CR8 reduced the number of TUNEL-positive cell by 40% of compared to vehicle group (Figure 11 D–E). In the R2 and C2 regions CR8 reduced the number of TUNEL-positive cells by 66% compared to control (Figure 11 E, p<0.05 vs vehicle). The strongest protective effect was observed in R4 and C4 regions, where CR8 afforded a 3- or 4-fold (p<0.05) reduction in the number of TUNEL-positive cells compared to vehicle-treated animals (Figure 11 E).

Finally, stereological assessment of surviving neurons (NeuN^+^/DAB^+^ cells) was also examined at 5 weeks after SCI. SCI resulted in 63% neuronal cell loss in spinal cord sections spaced 1 mm apart from 5 mm caudal to 5 mm rostral the injury epicenter (419,718±5,2581 vs. 113,5551±38,980 NeuN^+^ cells for vehicle and sham samples respectively). CR8 significantly improved neuronal survival at 5 weeks after injury, as compared to vehicle-treated samples (Figure 11 F, 66,2791±59331 vs 41,9718±52581 NeuN^+^ cells for CR8-treated and vehicle-treated samples respectively, p<0.05). There was no difference in NeuN^+^ cells between sham and CR8- treated sham groups. Thus, our data demonstrate the neuroprotective effects of CR8 following experimental SCI.

## Discussion

In the present study, we describe the pro-apoptotic up-regulation of the E2F1/CDK1 pathway in injured spinal cord neurons, as well as in two different cell culture models using primary cortical neurons. Furthermore, we demonstrate the neuroprotective effects of inhibition of E2F1/CDK1 signaling *in vitro*, using either selective shRNAs or selective CDK inhibitors, as well as in a clinically relevant rat contusion model of SCI using a novel and selective CDK inhibitor.

E2F1 transcriptional activity has been implicated in neuronal apoptosis as a common component of cell cycle re-activation dependent death signaling. E2F1 either promotes or induces neuronal apoptosis in various *in vitro* models- including activity deprivation in cerebellar granule neurons, kainic acid neurotoxicity, oxygen/glucose deprivation and ß-amylod induced apoptosis [36]–[39]. Our results indicate that E2F1 is sufficient to induce neuronal apoptosis, as well as necessary for trophic deprivation induced neuronal death. Up-regulation of this transcription factor in apoptotic motor neurons after SCI expands the list of E2F1 driven apoptotic models. Although, several pro-apoptotic mechanisms, including p53 transactivation, have been described downstream of E2F1, CDK1 induction appears to represent an important pathway for E2F1 mediated neuronal apoptosis [22]. Our data show a rapid and sustained elevation of E2F1 protein levels whose peak at 2 h following SCI precedes the CDK1 expression peak at 5 h post-SCI. This expression pattern is consistent with the hypothesis that SCI up-regulates E2F1, which in turn determines CDK1 up-regulation. Furthermore, we demonstrate that shRNA-mediated knockdown of E2F1 expression causes down-regulation of CDK1 expression. These data provide additional support to the proposed model where E2F1 is a key regulator of CDK1 expression and SCI-induced overexpression of E2F1 is at least in part responsible for the CDK1 elevation. In this study we not only show that up-regulation of E2F1 is followed by induction of CDK1 but also by increase levels of cyclin A and cyclin B1 in injured spinal cord. Whereas accumulation of cyclin B1 has been shown in compromised neurons [40], in human neurodegenerative conditions [41]–[43] and in animal models of injury [44], [3] the increase of cyclin A protein during neuronal injury has not been previously described. Although both of these cyclins are capable of CDK1 binding and activation, the role of these proteins in neuronal apoptosis has not yet been clearly defined.

SCI induces increased CDK activity, including CDK1 specific phosphorylation at Ser54-n-myc and expression of cyclin B1, which is the major co-activator of CDK1. Together, these data indicate activation of CDK1 signaling following SCI. It has been recently shown that CDK1, in complex with cyclins A and B1, specifically mediates n-myc Ser54 phosphorylation in primary cerebellar granule neuron precursors [45]. Pro-apoptotic CDK1 activation has been described in cerebellar granule neurons when apoptosis is induced by activity deprivation [22]–[23]. In this study we demonstrate co-localization of E2F1 with caspase-3 in injured spinal cord, as well as increased CDK1 immunofluorescence in spinal cord neurons undergoing apoptosis. Unlike E2F1, CDK1 expression was not sufficient to trigger apoptosis in cultured neurons. However, our data show that overexpression of CDK1 results in a significant enhancement of trophic deprivation-dependent neuronal death. Furthermore, the effects of shRNA-mediated down-regulation of CDK1 here underscore the requirement of this protein kinase for trophic deprivation-induced death of cortical neurons. Previous studies have used a dominant negative mutant of CDK1 to protect cerebellar granule neurons from low potassium induced apoptosis; the same CDK1 mutant also protected against wild type Bad or E2F1 transcription factor expression induced neurotoxicity [22]. Thus, our new data confirm and extend previous research and indicate that inhibition of either E2F1 or CDK1 enhances primary cortical neuronal survival under apoptotic conditions and implicate activity of these proteins in neuronal death after spinal cord injury.

Roscovitine, flavopiridol and several other small molecule inhibitors of CDKs have been extensively studied using *in vitro* and *in vivo* models of neuroprotection [46], [15]–[16], [26]. Roscovitine (also known as CYC202 or seliciclib) is a 2,6,9-tri-substituted purine that is in late phase 2 trials for non-small cell lung cancer and nasopharyngeal cancer [47]–[49]. In our previous studies, Roscovitine mediated attenuation of cell cycle re-activation, progressive neurodegeneration and neurological dysfunction in several models of TBI [16], [28]. However, roscovitine has not been evaluated for its potency to inhibit E2F1/CDK1 pathway. Here we show that CR8, which is purine analogue that is a more potent inhibitor of CDK1 than roscovitine [50] reduced biochemical markers of apoptosis, such as cleavage of the caspase-3 and α-fodrin, as well as afforded strong neuroprotection in trophic deprived or campthotecin-treated post-natal cortical neurons. CR8 provided neuroprotection at concentrations 10 or 50 times less than roscovitine in trophic deprived or campthotecin treated neurons. CR8 reversed up-regulation of E2F1, cyclins A and B1, significantly attenuated the increase in CDK activity- phospho-(Ser54) n-myc levels- and reduced neuronal apoptosis in the injured spinal cord. Although inhibition of n-myc phosphorylation at Ser 54 may reflect a direct inhibition of CDK1 by CR8, the potential mechanisms for the reduction in E2F1, as well as down-regulation of cyclins A and B1 protein levels are less clear. Our results indicate that CR8-mediated inhibition of yet unidentified pro-apoptotic targets may interfere with E2F1/CDK1 pathway at multiple levels to block pro-apoptotic signaling down-stream, as well as up-stream of E2F1. Of note, CR8 may also target CDK5, CDK7 and CDK9, and perhaps inhibition of these protein kinases contributes to the strong anti-apoptotic effect of this drug. Therefore, further investigations are required to address the detailed mechanisms of CR8-mediated neuroprotection.

We have also found that the SCI-induced accumulation of the 145/150 kDa cleavage products of α-fodrin, caspase-3 activation and TUNEL-positive neurons in the injured cord were significantly attenuated by CR8. Fodrin, a high molecular weight (240 kDa) cytoskeletal protein present in cell membranes, undergoes degradation catalyzed by activated caspases and other proteases during apoptosis [51]–[53]. The cleavage of α-fodrin, which continues for up to 7 days leads to membrane malfunctions, cell shrinkage and may reflect widespread damage and remodeling in injured spinal cord. Notably, CR8 treatment following SCI results in a late (five weeks) increase in neuronal survival.

Aberrant cell cycle activation induces proliferation in mitotic cells such astrocytes and microglia. Administration of cell cycle inhibitors- including flavopiridol, roscovitine and olomoucine- inhibits microglial and astrocyte proliferation both *in vitro* and in vivo [2], [15]–[16], [26]. Our previous studies have indicated that sustained microglial activation after CNS trauma may play a role in neuronal cell loss following the release of neurotoxic molecules such as NO [2], [15], [26], [28]. Our recent *in vitro* results demonstrate that CR8 exhibits a 10–20 fold higher potency than roscovitine in suppression of proliferation and activation of cultured primary microglia (unpublished data). Thus, the robust effect of CR8 on reactive microglia might reflect an important component of the overall CR8-induced neuroprotection.

In conclusion, we provide evidence that inhibition of E2F1/CDK1 signaling pathway confers neuroprotection both *in vitro* and after rat spinal cord injury. CR8 appears to be a potent inhibitor of this pathway and early treatment reduces neuronal cell loss chronically after SCI. The latter observations provide additional support for the neuroprotective action of cell cycle inhibitors in experimental SCI and indicate that this may be an effective therapeutic target for the clinical disorder.
